# Impact of Erythromycin as a Prokinetic on the Gut Microbiome in Children with Feeding Intolerance—A Pilot Study †

**DOI:** 10.3390/antibiotics12111606

**Published:** 2023-11-08

**Authors:** Aravind Thavamani, Senthilkumar Sankararaman, Hilmi Al-Shakhshir, Mauricio Retuerto, Sujithra Velayuthan, Thomas J. Sferra, Mahmoud Ghannoum

**Affiliations:** 1 Division of Pediatric Gastroenterology, Hepatology & Nutrition, Department of Pediatrics, UH Rainbow Babies and Children’s Hospital, Cleveland, OH 44106, USA; aravind.thavamani@uhhospitals.org (A.T.); sujithra.velayuthan@nationwidechildrens.org (S.V.); thomas.sferra@uhhospitals.org (T.J.S.); 2 Department of Pediatrics, Case Western Reserve University School of Medicine, Cleveland, OH 44106, USA; 3 Department of Radiology and Imaging Sciences, Emory School of Medicine, Atlanta, GA 30307, USA; halshak@emory.edu; 4 Department of Radiology and Imaging Sciences Atlanta VA Medical Center, Decatur, GA 30033, USA; 5 Center for Medical Mycology, Department of Dermatology, Case Western Reserve University School of Medicine, Cleveland, OH 44106, USA; mar153@case.edu (M.R.); mag3@case.edu (M.G.); 6 Division of Pediatric Neurogastroenterology and Motility, Department of Pediatrics, Nationwide Children’s Hospital, Columbus, OH 43205, USA; 7 Department of Dermatology, University Hospitals Cleveland Medical Center, Cleveland, OH 44106, USA

**Keywords:** gut microbiome, bacteriome, mycobiome, erythromycin, feeding intolerance

## Abstract

Background: Studies have demonstrated that the gut microbiome changes upon exposure to systemic antibiotics. There is a paucity of literature regarding impact on the gut microbiome by long-term usage of erythromycin ethyl succinate (EES) when utilized as a prokinetic. Methods: Stool samples from pediatric patients with feeding intolerance who received EES (*N* = 8) as a prokinetic were analyzed for both bacteriome and mycobiome. Age-matched children with similar clinical characteristics but without EES therapy were included as controls (*N* = 20). Results: In both groups, Proteobacteria, Firmicutes, and Bacteroidetes were the most abundant bacterial phyla. Ascomycota was the most abundant fungal phyla, followed by Basidiomycota. There were no significant differences in richness between the groups for both bacterial and fungal microbiome. Alpha diversity (at genus and species levels) and beta diversity (at the genus level) were not significantly different between the groups for both bacterial and fungal microbiome. At the species level, there was a significant difference between the groups for fungal microbiota, with a *p*-value of 0.029. We also noted that many fungal microorganisms had significantly higher *p*-values in the EES group than controls at both genera and species levels. Conclusions: In this observational case-control study, the prokinetic use of EES was associated with changes in beta diversity between the groups for mycobiome at the species level. Many fungal microorganisms were significantly higher in the EES group when compared to the controls. Confirmation of these results in larger trials will provide further evidence regarding the impact of EES on gut microbiota when utilized as a prokinetic agent.

## 1. Introduction

Antibiotics have been widely used in many sectors, such as livestock farms, veterinary medicine, agriculture, hospitals, and industries [1]. Since the discovery of penicillin by Alexander Fleming in 1928, antibiotics revolutionized medical management, saving millions of lives. However, the usage of antibiotics, specifically indiscriminate usage, can lead to collateral adverse effects, such as *Clostridioides difficule* infection, and the development of resistant organisms.

Both short- and long-term use of antimicrobials have a profound impact on the gut microbiome [2,3]. Evidence is also getting stronger that antibiotic exposure early in life with resulting gut dysbiosis can lead to the development of many chronic diseases such as overweight/obesity and untoward metabolic alterations [4,5,6,7]. Post-antibiotic gut dysbiosis is commonly characterized by decreased diversity, reduced abundance of beneficial species, increased abundance of potential pathobionts, and altered metabolomics [8,9,10,11,12]. Contrary to the popular notion of post-antibiotic dysbiosis, in very few selective circumstances, antibiotics may also have a positive modulation of the gut microbiome, referred to as eubiosis [13,14,15]. For example, rifaximin, a non-absorbable antibiotic that is being utilized in multiple clinical settings such as irritable bowel syndrome, hepatic encephalopathy, and small intestinal bacterial overgrowth, has been shown to exert beneficial effects with a positive effect on gut microbiota [14,15].

Macrolide antibiotics (erythromycin and azithromycin) at lower doses are often used off-label for their prokinetic action to enhance feeding tolerance in neonates and infants [16,17,18]. They act as a motilin agonist and initiate phase-III activity of migrating motor complexes (MMCs) in the upper gastrointestinal (GI) tract, resulting in enhanced GI motility and improved gastric emptying [19,20]. Given the challenges in the diagnosis of functional and motility GI disorders in infants and young children, prokinetic medications are often empirically used in children with severe feeding issues [21,22,23,24,25,26,27,28,29,30,31,32].

Most studies evaluating the impact of antibiotics on gut microbiota have been performed in adults and there is a paucity of pediatric literature [33,34,35]. Similarly, most studies focused on bacteriome, and studies on fungal community (mycobiome) remain sparse [33,34,35]. The mycobiome does not receive much attention, as fungi consist of only 0.1% of the entire gut microbes, and there are also challenges in their interpretation, such as high interpersonal and intrapersonal variability of mycobiome [36,37]. Unlike bacterial gut microbiota, the gut mycobiota has fewer taxa, and also lacks stability [38]. With the advent of the latest sequencing technologies and bioinformatic applications, more light has been shed on the composition and implications of gut mycobiome [36,39,40].

Infants with feeding intolerance have been noted to have altered gut microbiota [41,42]. Hu and colleagues noted that preterm infants with feeding intolerance had decreased microbial diversity, lower abundance of beneficial microbiota, and higher abundance of pathobionts compared to infants who tolerated their feeds well [41]. The long-term impact of prokinetic use of erythromycin on the gut microbiota in children with feeding intolerance remains elusive. Specifically, the data on the effect of erythromycin at prokinetic doses on mycobiome is sparse. We hypothesized that the long-term usage of EES as a prokinetic may result in an alteration in the diversity of bacterial and fungal microbiota. The objective of this pilot study was to evaluate the impact of prokinetic use of EES on gut microbiota (both bacterial and fungal microbiome) in children with feeding intolerance.

## 2. Results

Eight patients in the EES group and 20 in the control group were included after the quality control for the analysis. The clinical characteristics were similar and not significantly different between the EES and control groups (Table 1). Eight patients received EES for a duration ranging from 4–32 weeks (median 12 weeks, [IQR 9–23]).

**Figure 1:**
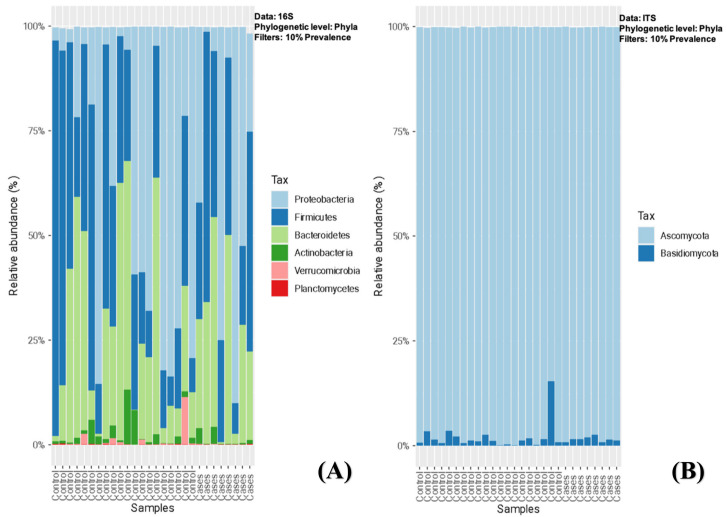
(**A**,**B**)-Relative abundance of sequence reads of the most abundant bacterial (**A**) and fungal phyla (**B**) using 16S rRNA (16S) and ITS sequencing, respectively.

On stool microbiome analysis, we compared the bacterial and fungal composition between the EES (cases) and control groups. We utilized 16S rRNA (16S) for bacteriome and internal transcribed spacer (ITS) sequencing for mycobiome. In both groups, Proteobacteria, Firmicutes, and Bacteroidetes were the most abundant phyla (filtered at 10% prevalence), followed by Actinobacteria and Verrucomicrobia, and there were no significant differences noted between the groups (Figure 1A). Among the mycobiota, Ascomycota was the most abundant fungal phyla, followed by Basidiomycota (one patient in the control group had a relatively higher abundance > 10%), and, similarly, no significant differences were noted between the groups (Figure 1B).

**Figure 2:**
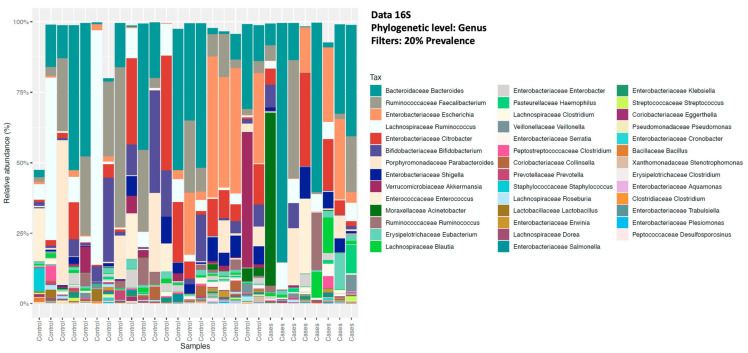
Relative abundance of sequence reads of the most abundant bacterial genera using 16S rRNA (16S) sequencing.

**Figure 3:**
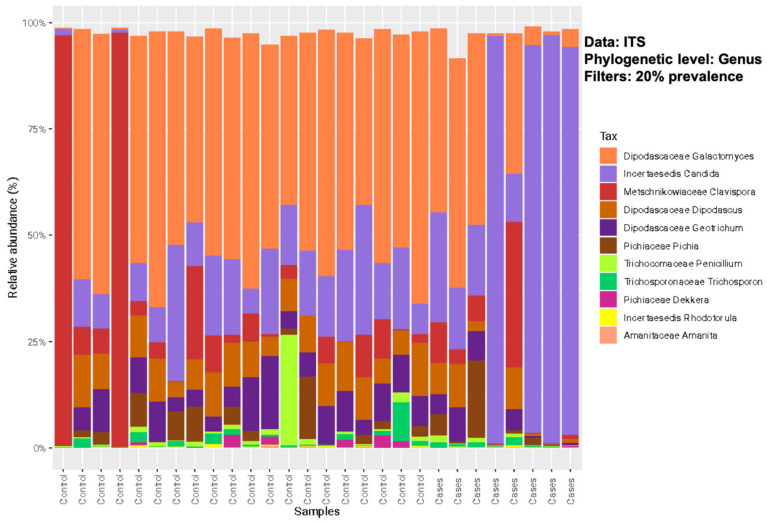
Relative abundance of sequence reads of the most abundant fungal genera using ITS sequencing.

The relative abundance of the common genera in both groups was depicted in descending order for bacterial microbiota and, overall, no significant differences were noted between the groups (Figure 2). At the bacterial genus level (filtered at 20% prevalence), *Bacteroides*, *Faecalibacterium*, *Escherichia*, *Ruminococcus*, and *Citrobacter* were the most abundant genera for both groups. Among the fungal genera, *Galactomyces*, *Candida*, and *Clavispora* were the abundant genera for both groups (Figure 3). Among the control group, *Galactomyces* was the most predominant genera, but, in the EES group, *Candida* dominated in 4 patients, followed by *Galactomyces* in 3 and *Clavispora* in 1.

**Figure 4:**
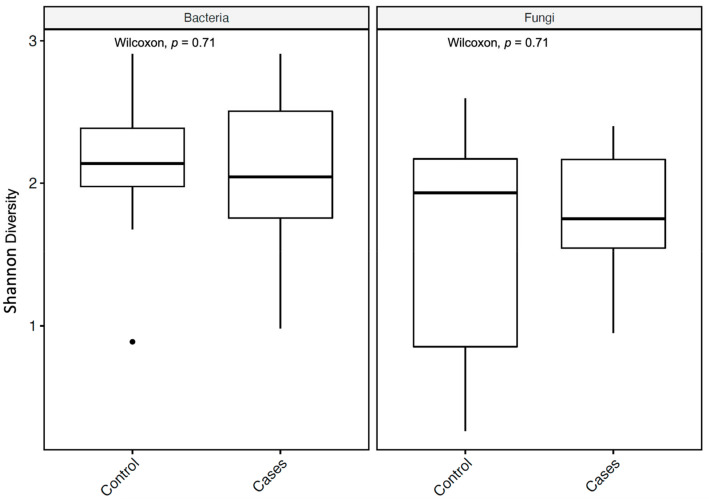
Alpha diversity metrics of bacteria and fungi, using the Shannon diversity index, for bacterial and fungal microbiota with *p*-values (Wilcoxon rank-sum test). In the box plots, the central rectangle spans the first to third quartiles (the interquartile range, IQR), the central line inside the rectangle shows the median, and the whiskers and dots indicate the variability outside the upper and lower quartiles.

The alpha diversity (measured by the Shannon diversity index) was not significantly different between the groups for both bacterial and fungal microbiota, with a *p*-value of 0.71 for both, utilizing the Wilcoxon rank-sum test (Figure 4).

**Figure 5:**
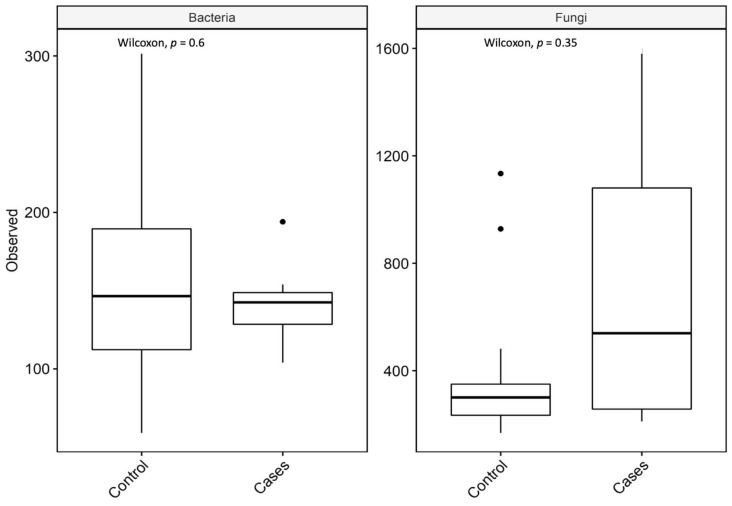
Observed taxon richness index (observed) for bacterial and fungal microbiota with *p*-values (Wilcoxon rank-sum test). In the box plots, the central rectangle spans the first to third quartiles (the interquartile range, IQR), the central line inside the rectangle shows the median, and the whiskers and dots indicate the variability outside the upper and lower quartiles.

For richness, the observed taxon richness index was utilized and it was not significantly different for both groups; *p*-values were 0.6 and 0.35 (Wilcoxon rank-sum test) for bacterial and fungal microbiota, respectively (Figure 5).

**Figure 6:**
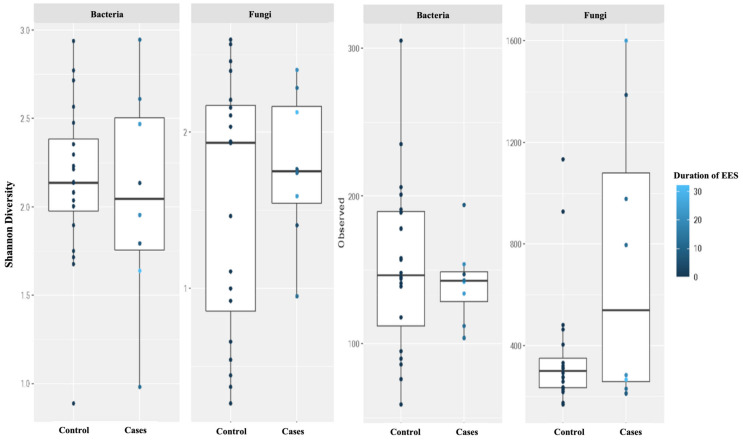
Alpha diversity was measured by the Shannon diversity index (Shannon) and the richness was measured using the observed taxon richness index (Observed) for both bacteria and fungi. Duration (in weeks) of erythromycin ethylsuccinate therapy was noted with color change. In the box plots, the central rectangle spans the first to third quartiles (the interquartile range, IQR), the central line inside the rectangle shows the median, and the whiskers and dots indicate the variability outside the upper and lower quartiles.

Further, we did not see a correlation between diversity and richness and the duration of EES therapy (Figure 6).

**Figure 7:**
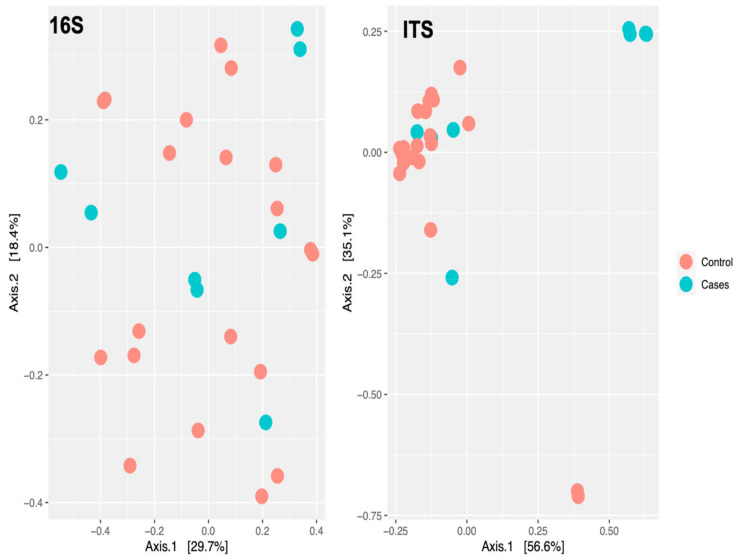
Multidimensional scaling plot of bacteriome (noted as 16S for 16S rRNA sequencing) and mycobiome (noted as ITS for ITS sequencing) at the genus level by erythromycin ethylsuccinate therapy status.

**Figure 8:**
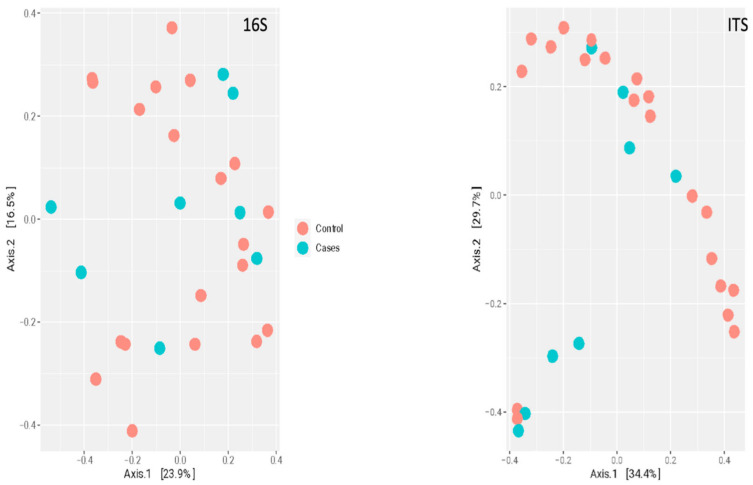
Multidimensional scaling plot of bacteriome (noted as 16S for 16S rRNA sequencing) and mycobiome (noted as ITS for ITS sequencing) at the species level by erythromycin ethylsuccinate therapy status.

At the genus level, there were no clear clustering patterns noted in the multidimensional scaling (MDS) for both bacteriome and mycobiome between the two groups (Figure 7). The beta diversity was calculated by PERMANOVA. At the species level, there was no significant difference between the participants (*p*-value of 0.7129) based on the EES therapy for bacteriome. Meanwhile, a *p*-value of 0.0297 was noted for the mycobiome at the species level, indicating a significant difference between the two clusters based on EES (Figure 8).

**Figure 9:**
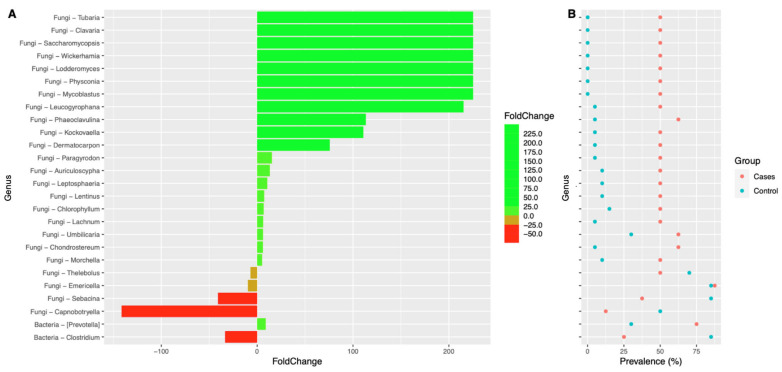
Colplot demonstrating significantly different microbiota between fungal and bacterial composition for both cases and controls at the genus level. In this figure, (**A**) provides the fold change after the erythromycin ethylsuccinate and (**B**) denotes the changes in the prevalence.

**Figure 10:**
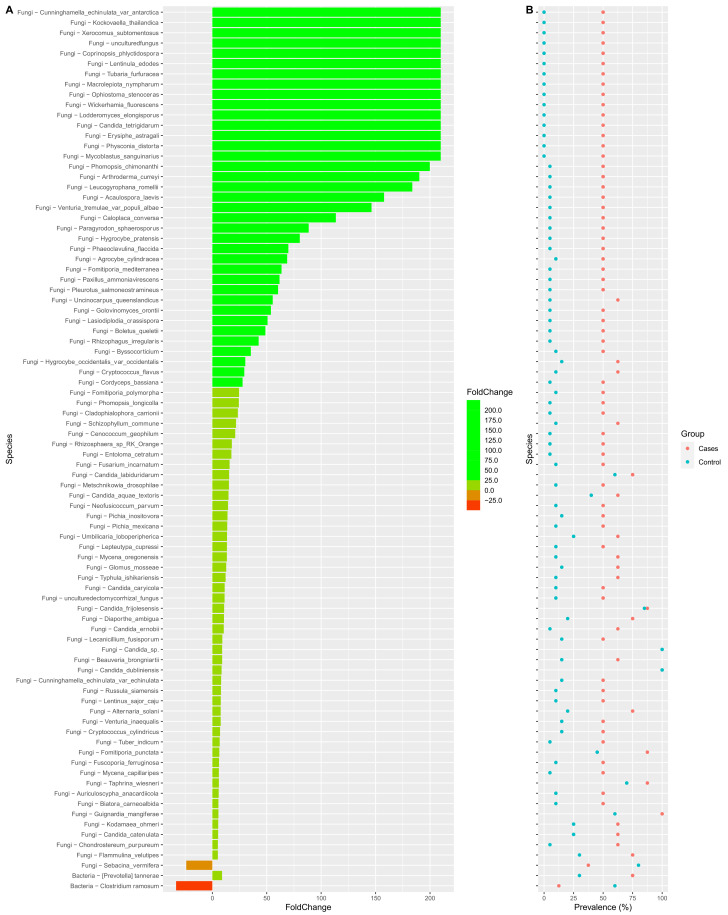
Colplot demonstrating significantly different microbiota between fungal and bacterial composition for both cases and control groups at the species level. In this figure, (**A**) provides the fold change after the erythromycin ethylsuccinate and (**B**) denotes the changes in the prevalence.

Interestingly, we also noted that the prevalence of many fungal genera were significantly higher in the EES group when compared to the control group (Figure 9). A very similar trend was also noted at the species level (Figure 10). Supplementary Files S1 and S2 depict the fold change of genera and species, respectively, along with their prevalence. *p*-values were also noted between the EES and control groups. At the genus level, the prevalence and relative abundance of *Prevotella* increased with EES usage and, meanwhile, for *Clostridium*, the prevalence and relative abundance decreased (Figure 9 and Supplementary File S1). For the fungal microbiota, the prevalence of many genera increased (Figure 9 and Supplementary File S1). The most prevalent (100%) genera in both groups included *Galactomyces*, *Candida*, and *Rhizopogon* (Supplementary File S1). Interestingly, with the EES exposure, the mean relative abundance of *Galactomyces* and *Rhizopogon* decreased, and the mean relative abundance of *Candida* increased (Supplementary File S1). Many species of *Candida* were widely prevalent in both groups, and the mean relative abundance significantly increased with exposure to EES (Figure 10 and Supplementary File S2).

## 3. Discussion

This prospective, observational, case-control study is the first of its kind to evaluate the effects of long-term use of EES as a prokinetic in pediatric patients with feeding intolerance. The median duration of EES therapy was 12 weeks (range 4–32 weeks), which allowed us to evaluate the long-term effects. We noted that diversity (both alpha and beta) and richness were not significantly different between EES and control groups for bacteriome. For mycobiome, the alpha diversity, beta diversity (only at the genus level), and richness were not significantly different between the groups. We did not observe an association between the duration of EES therapy and diversity and richness in both bacteriome and mycobiome. Further, we noted an increase in the relative abundance and prevalence of many fungi, both at species and genus levels, with EES exposure.

The impact of antibiotics on the gut microbiome is complex and variable, based on multiple factors such as patient characteristics and antibiotic details [43]. The majority of the prior studies evaluated short-term (<1–2 weeks) treatment with macrolides and its implications on the gut bacteriome. A recent meta-analysis summarized various clinical trials and concluded that short-term exposure to azithromycin was associated with significantly reduced alpha diversity in the gut bacteriome of children [44]. The exposure to macrolides correlated with decreased richness for twice the duration when compared with penicillin therapy [44]. On the contrary, our study did not show significant differences in diversity (both alpha and beta) and richness between the groups for bacteriome. Further, various investigators demonstrated that short-term exposure to systemic antibiotics were associated with decreased gut bacterial relative abundance [35,44]. We noted an increased prevalence and relative abundance of *Prevotella* with EES usage, and, meanwhile, for *Clostridium*, the prevalence and relative abundance decreased. Animal experiments serve as excellent models to evaluate the effects of systemic antibiotics. In mice, exposure to erythromycin or penicillin G, or a mixture of both, swiftly resulted in altered microbial composition and took a long time to revert to preexposure composition [45].

Low-dose, long-term macrolide antibiotics are often utilized in chronic respiratory disorders for their anti-inflammatory action, and their impact on oropharyngeal and respiratory microbiome has been previously documented. Burr et al. investigated the impact of long-term (4-week) exposure to EES and azithromycin therapies on oropharyngeal microbiome [46]. Both antibiotics did not alter the respiratory microbial composition significantly, which is very similar to the results of our study.

In our study, Proteobacteria, Firmicutes, and Bacteroidetes were the most abundant phyla, and no significant differences were noted between the groups. Interestingly, we found an increased relative abundance of Proteobacteria in both groups, which has been previously reported in young children with functional GI disorders and patients with many systemic diseases [47,48]. The increased abundance of Proteobacteria in the EES group could be related to chronic exposure to EES or also could be a sign of dysbiosis, which has been documented in many disease states [49,50,51,52]. Using Korea native ricefish (*Oryzias latipes*), chronic exposure to minimal concentrations of EES and ampicillin demonstrated significantly increased Proteobacteria and reduced Fusobacteria [53].

Similar to our study results, Firmicutes, Bacteroidetes, Fusobacteria, Proteobacteria, and Actinobacteria were the most abundant phyla in oropharyngeal microbiome of people with asthma, and there were no significant differences between participants who received azithromycin or a placebo [54]. On the contrary, Taylor and colleagues documented that exposure to azithromycin did not change the airway bacterial load but significantly reduced the bacterial diversity and altered the composition of *Haemophilus influenzae* noted compared to the placebo [55]. Segal and colleagues noted alterations in the respiratory microbiome upon exposure to azithromycin [56]. Here, in patients with emphysema, azithromycin treatment for eight weeks did not change the bacterial burden but did decrease the alpha diversity when compared to the placebo [56].

These variations in inferences between different microbiome studies are likely due to differences in the methodology utilized, different doses and durations of antibiotics, and also due to the differences in the underlying patient population [35]. Another possible reason for the lack of notable differences in diversity and richness between the groups in our study might be related to the smaller sample size.

In the present study, *Bacteroides*, *Faecalibacterium*, *Escherichia*, *Ruminococcus*, and *Citrobacter* were the most abundant genera for both groups, and no significant differences were noted in bacteriome. In other words, we did not find any specific bacterial signatures identified in our patient population compared to prior reported pediatric studies [57,58,59].

Antibiotics may have a profound impact on fungal microbiota and their effects could be longer lasting compared to bacteriome changes [60]. In our study, Ascomycota and Basidiomycota were the dominant phyla in both groups and no significant differences were noted between the groups. Prior published studies have noted similar preponderance of these phyla in both children and adults [33,38,61,62]. Among the fungal genera, *Galactomyces*, *Candida*, and *Clavispora* were the abundant genera for both groups. Among the control group, *Galactomyces* was the most predominant genera, but in the EES group, *Candida* dominated, followed by *Galactomyces.* Even though *Candida* is part of normal human mycobiota, its expansion has been noted in many inflammatory conditions [63,64,65,66,67,68]. The effect of antibiotics (amoxicillin and/or macrolides) on gut mycobiome was studied in infants by Ventin-Holmberg and colleagues in a longitudinal fashion [69]. Here, the antibiotic-treated group demonstrated significantly higher fungal diversity and richness. In the present study, the mycobial diversity (only at the genus level) and richness were not significantly different between the groups. At the species level, the beta diversity for EES and control groups was significantly different (*p*-value—0.0297). This difference is likely due to the smaller sample size in the EES group rather than true clustering patterns noted between the groups (Figure 8).

In our study, we noted an increase in the relative abundance and prevalence of many fungi, both at the species and genus levels, with EES exposure. Generally, the commensal bacteria predominate in gut microbiota and mitigate the fungal overgrowth and invasive fungemia [70]. When the bacteria are perturbed by antibacterial antibiotics, opportunistic fungal (especially *Candida)* growth may increase and, at times, could predispose the host to systemic fungal infections [34,69,71]. Prior culture studies have substantiated a drastic increase in *Candida* following ingestion of antibiotics [72,73]. Various reasons, such as increased nutrient availability and modifications of growth conditions, are responsible for this proliferation of fungi [69,72,73]. Bacterial metabolites (e.g., short-chain fatty acids such as butyrates) have also been documented to inhibit the growth of many pathogenic fungi under normal circumstances [34,74,75]. In a murine model, administration of systemic antibiotics for three days decreased cecal anaerobic bacteria and increased enteric bacilli [76]. When investigators challenged the mice with oral administration of *Candida albicans,* decreased cecal anaerobic bacteria correlated with intestinal mucosal surface adhesion, colonization, and further dissemination of fungi from the GI tract, underscoring the relationship between bacterial and fungal microbial changes upon exposure to antibiotics [76].

We recognize several limitations of this study. This pilot study involved a small sample in a single center, which affects the generalizability of the results. Studies with small sample sizes are inevitable in pediatric microbiome studies focusing on a specific patient population, such as ours [69]. Also, the strict inclusion criteria, which aimed to minimize known confounding factors impacting the gut microbiome, drastically reduced the number of recruits. The ideal way to evaluate the promotility effects of EES is by conducting a randomized controlled trial (RCT), or at least by a prospective cohort study design comparing the gut microbiome pre- and post-exposure to EES in a prolonged longitudinal fashion. As the decision of EES therapy was determined entirely by the clinical team, we did not have access to the pre-exposure samples.

The definition of feeding intolerance was also entirely clinical, which might have introduced some confounding in the recruitment of controls. Also, other important clinical characteristics known to influence the gut microbiome, such as delivery method, gestational age, diet (history of breastfeeding vs. formula), and neurodevelopmental status were not evaluated between the groups. As the majority of participants were less than three years of age, the normal age-dependent maturation of the microbiota might have confounded our interpretation. We recruited age-matched controls to minimize this confounding factor. Further, we did not recruit age-matched healthy children as controls. Under ideal circumstances, age-matched healthy children may better serve as controls and might have demonstrated a healthy microbial pattern.

Despite the above limitations, we have numerous strengths as well. This prospective observational pediatric study is the first of its kind to evaluate the effects of long-term effects EES as a prokinetic in patients with feeding intolerance. The median duration of EES therapy was 12 weeks (range 4–32 weeks), which allowed us to evaluate the long-term effects. Our strict inclusion criteria helped us to minimize many confounders. Additionally, we also recruited controls with similar clinical characteristics who were age-matched for the study population in an approximately 2:1 ratio. Further, we analyzed the mycobiome in addition to the bacteriome. Mycobiome studies in pediatrics remain very sparse and this study provides useful insights into the gut microbiota in children on long-term EES. Further prospective studies with larger samples are needed to elucidate the changes in gut bacteria and fungi and their complex interactions after long-term use of EES as a prokinetic [77,78].

## 4. Materials and Methods

This prospective observational case-control study was conducted at the University Hospitals (UH) Rainbow Babies & Children’s Hospital and Case Western Reserve University, Cleveland, Ohio and the protocol was approved by the Institutional Review Board (study number 20200634).

Inclusion criteria: Potential study participants between 2 months and 18 years of age were prescreened and identified at clinic visits for eligibility. Children with feeding intolerance who were already prescribed EES for at least four weeks for prokinetic indication constituted the EES group (also referred to as the cases). Eleven patients on EES were successfully enrolled in this group. The decision to initiate EES therapy was entirely decided by the primary pediatric gastroenterologist without input from the research team. Gastroparesis (either confirmed or suspected) was the indication for initiating EES. Gastroesophageal reflux disease, cow milk allergy, constipation, and aspiration of feeds were comorbid diagnoses. Twenty-two age-matched patients with feeding intolerance with similar clinical profiles but not on EES were recruited as controls.

Exclusion criteria: Patients with a recent episode of bacterial or viral illness in the preceding 12 weeks were excluded. Also, patients who received antibiotics other than EES and/or exposure to probiotics in the preceding 12 weeks were excluded to minimize confounding effects on gut microbiota. Patients with known immunodeficiency disorders, malignancies, systemic inflammatory disorders, autoimmune conditions, and mucosal GI disorders were also excluded.

Participants were enrolled after informed consent from their parents or legal guardians. An assent was obtained from children between 7–18 years of age. A stool sample kit (BD BBL™ CultureSwab™ EZ, Beckton Dickinson, Franklin Lakes, NJ, USA) was provided, along with instructions, to the participant’s families. Stool specimens were collected in a consistent way to minimize confound effects and were stored in the Integrated Microbiome Core Laboratory at the Case Western Reserve University and University Hospitals Cleveland Medical Center, Cleveland, OH, USA.

### 4.1. Sample Collection and Processing

All collected stool samples were instantly placed in FastPrep^®^ tubes (MP Biomedicals™, Cat# 5076-200-34340, Solon, OH, USA) containing 500 μL of glass beads (Sigma-Aldrich G8772-100g, St. Louis, MI, USA) and 1 mL ASL™ lysis buffer (Qiagen DNA Extraction Kit, Hilden, Germany). To reduce batch effect, all samples were stored at −20 °C, then processed and analyzed concurrently for microbial composition. The methodology, including DNA extraction, PCR amplification, library preparation, and sequencing, is detailed in Appendix A and was previously reported by our lab [79,80]. Library sequencing was completed on an Ion Torrent S5 sequencer (ThermoFisher Scientific,. Waltham, MA, USA). Analysis of barcode-sorted samples was performed in a custom pipeline based on Greengenes V13_8 and Unite V7.2 databases illustrated for taxonomic classification of 16S rRNA and ITS sequences, respectively. Sequencing reads were clustered into operational taxonomic units (OTUs, 3% distance), described by community metrics, and classified within the Qiime 1.8 taxonomy bioinformatics pipeline.

### 4.2. Bioinformatic Analysis

Raw data of the microbial (both 16S and ITS data) read counts were loaded to R version 4.0.3. Sample metadata was further processed using R-package microbiome 1.12.0 and phyloseq 1.34.0, a phyloseq object with a read count matrix (species-level identification), taxonomic table (kingdom to species), and metadata, was generated. When processing 16S (for the bacteriome) and ITS (for the mycobiome) data, each of them was processed separately and later combined after the read counts were normalized and transformed to relative abundance.

### 4.3. Pre-Processing and Quality Control

Before creating the phyloseq object for the 16S and ITS data, the taxonomic table was evaluated to eliminate species and phyla which were annotated as “unidentified” or lacking identification at a species level with an annotation “empty cell”. This elimination ensured only identifiable species were utilized in the bioinformatic analysis. With regards to total read counts per sample, a cutoff of 500 read counts was utilized as the minimum needed read count. For the final analysis, only 8 samples in the EES group and 20 in the control group were utilized after this quality control.

### 4.4. Data Exploration and Data Analysis

Separate composition bar graphs were developed on 16S and ITS relative abundance after aggregating the data to the phyla level and filtering on phyla prevalence of 10% amongst all samples. Ordinate analysis/principal component analysis (PCA) was performed on bacterial microbiome and mycobiome data separately, after aggregating data to the phyla level as a means to reduce complexity. Ordinate analysis was performed using the “ordinate” function part of microbiome R-package version 1.12.0, with this method utilized as “multidimensional scaling” (MDS) and distances using the “Bray–Curtis” method for beta diversity. Bar plots were created using 16S and ITS based on relative abundance. The data were first filtered on species with a prevalence of 20% within all samples, and then the corresponding phyloseq objects were merged.

Given the nonparametric nature of the data, the Wilcoxon rank-sum test was utilized to evaluate for significantly different species between the two groups, and the foldchange was measured using the mean relative abundance of a species within each group, utilizing the foldchange function from the R-package. Box plots were constructed to estimate diversity and richness. In the box plots, the central rectangle spans the first to third quartiles (the interquartile range, IQR), the central line inside the rectangle shows the median, and the whiskers above and below the box indicate the variability outside the upper and lower quartiles. Alpha diversity was estimated using the Shannon diversity index and richness was measured using the observed taxon richness index.

## 5. Conclusions

In this pilot study, long-term exposure to EES treatment at the prokinetic dosage did not have significant changes in alpha diversity and richness in either the bacteriome or mycobiome in children with feeding intolerance. We noted that the relative abundance and prevalence of many fungal genera were significantly higher in the EES group when compared to the controls. A very similar trend was also noted at the species level. Prospective clinical trials with larger samples are needed to confirm or refute our preliminary data in this clinical setting.

## Figures and Tables

**Table 1:** Demographic Characteristics of the Cohort.

Variables at the Time of Enrollment	EES Group (*N* = 8)	Control Group (*N* = 20)	*p*-Value
Age (in months)	20 (14, 42)	18 (9, 27)	0.28
Gender			
Female	4 (50%)	5 (25%)	0.20
Male	4 (50%)	15 (75%)	
Weight (in Kg)	11.2 (9.1, 13.6)	9.8 (8.6, 11.2)	0.42
Length/height (in cm)	82 (73, 100)	79 (71, 86)	0.33
Malnutrition	1 (12%)	3 (15%)	0.17
Acid suppression therapy	4 (50%)	6 (30%)	0.10
Length of EES therapy (in weeks)			
4	1 (12%)		
8	1 (12%)		
12	3 (38%)		
20	1 (12%)		
24	1 (12%)		
32	1 (12%)		

Data were provided either as *N* (%) or Median (IQR), EES–erythromycin ethylsuccinate.

## Data Availability

Most of the data generated or analyzed during this study are included in this published article (Main document, Supplementary Materials, and Appendix A). Remaining data is available from the corresponding author.

## Supplementary Materials

The following supporting information can be downloaded at: https://www.mdpi.com/article/10.3390/antibiotics12111606/s1; File S1—Colplot demonstrating significantly different microbiota between fungal and bacterial composition for both cases and controls at the genus level. The Wilcoxon rank-sum nonparametric test was used to test for significantly different genera between patients in the EES group and the control group, and the fold change was calculated using the mean relative abundance of a genus within each group using the foldchange function from the R-package. Box plots were generated for all the significantly different genera; File S2—Colplot demonstrating significantly different microbiota between fungal and bacterial composition for both cases and controls at the species level with Wilcoxon rank-sum nonparametric test. Foldchange was calculated using the mean relative abundance of a species within each group using the foldchange function from the R-package.

## Appendix A. Detailed Explanation of DNA Extraction, PCR Amplification, and Sequencing

### Appendix A.1. DNA Extraction

DNA was extracted using the QIA amp Fast DNA stool mini kit (Qiagen GmpH, Hilden, Germany) per the manufacturer’s instructions. Samples were pelleted by centrifuge and placed in tubes with 1 mL of InhibitEX lysis buffer. Stool swabs were incubated for 60 min at 75 °C and further processed with Fastprep^®^ 96 two times for five minutes at 1800 RPM. Equal quantities of 100% ethyl alcohol and lysate were added together and passed over HiBind DNA Mini Columns (Omega Bio-tek, Norcross, GA, USA). DNA pellets were eluted with 50 μL molecular-grade water.

### Appendix A.2. PCR Amplification

Amplification of microbial 16S and 5.8S rRNA genes were performed using 16S-515 (5′-GGA CTA CCA GGG TAT CTA ATC CTG-3′) and 16S-804 (5′-(TCC TAC GGG AGG CAG CAGT-3′), ITS1 (5′-(TCC GTA GGT GAA CCT GCG G-3′), and ITS4 (5′-TCC TCC GCT TAT TGA TAT GC-3′) primers, respectively [81,82]. Q5 High-Fidelity master mix was used for PCR at a 1X concentration, along with twice the volume of molecular-grade water and 5 μL 100 mM of each primer. Then, 1.5 μL of undiluted DNA was added to each 50 μL reaction. Thermocycling conditions consisted of an initial denaturation (3 min at 98 °C), followed by 30 cycles of denaturation (10 s at 98 °C), annealing (10 s at 55 °C for the 16S primers and 20 s at 58 °C for the ITS primers), and extension (10 s at 72 °C), followed by a final extension process of 3 min 72 °C. Ten μL of each PCR product was extracted utilizing gel electrophoresis on 1.5% agarose gel (containing 7 μg/mL ethidium bromide).

### Appendix A.3. Library Preparation and Sequencing

The same quantities of extracted microbial reaction products were pooled and cleaned using AMPure XP beads (Beckman Coulter, Brea, CA, USA) to eliminate unutilized primers. Utilizing the Ion Plus fragment library kit (ThermoFisher Scientific, Waltham, MA, USA), pooled amplicons were exposed to end-repair enzyme for a period of 20 min at normal room temperature. Following a second AMPure cleanup, ligation of Ion Torrent P1 and unique barcoded ‘A’ adaptors for each pooled sample was carried out at 25 °C for 30 min. After AMPure removal of remaining adaptors, 10 μL of each barcoded sample was pooled and mixed in a single tube. Pooled library samples were further concentrated to reduced volume (25% of initial volume) using a Labconco vacuum for 1 h, along with heating.

The concentrated pool was then size-selected for the anticipated base-pair range of both 16S and ITS (300–800 bp) using Pippin Prep (Sage Bioscience, Beverly, MA, USA). Amplification of the size-selected library using platinum PCR SuperMix High Fidelity (ThermoFisher Scientific, Waltham, MA, USA) and the provided Ion Torrent Library amplification primer was done with conditions of 3 min denaturation at 95 °C, 7 cycles of denaturation at 95 °C for 15 s, annealing at 58 °C for 15 s, and extension at 70 °C for 90 s, and a final extension step of 5 min at 70 °C. Subsequent amplified library was then quantified using the Ion Library TaqMan Quantification Kit (Applied Biosystems by Life Technologies) on StepOne qPCR instrument. A dilution of 300 pM was added into IonSphere templating reaction on the Ion Chef.

Library sequencing was completed on an Ion Torrent S5 sequencer (ThermoFisher Scientific, Waltham, MA, USA). Analysis of barcode-sorted samples was performed in a custom pipeline based on Greengenes V13_8 and Unite V7.2 databases illustrated for taxonomic classification of 16S rRNA and ITS sequences, respectively. Sequencing reads were clustered into operational taxonomic units (OTUs, 3% distance), described by community metrics, and classified within the Qiime 1.8 taxonomy bioinformatics pipeline.
